# The influence of oral contraceptives on the exercise pressor reflex in the upper and lower body

**DOI:** 10.14814/phy2.16144

**Published:** 2024-07-11

**Authors:** T. J. Pereira, H. Edgell

**Affiliations:** ^1^ School of Kinesiology and Health Science York University Toronto Ontario Canada; ^2^ Muscle Health Research Centre York University Toronto Ontario Canada

**Keywords:** Hemodynamics, Mechanoreflex, Metaboreflex, Oral contraceptives, Ventilation

## Abstract

Previous research has demonstrated that oral contraceptive (OC) users have enhanced cardiorespiratory responses to arm metaboreflex activation (i.e., postexercise circulatory occlusion, PECO) and attenuated pressor responses to leg passive movement (PM) compared to non‐OC users (NOC). We investigated the cardiorespiratory responses to arm or leg metaboreflex and mechanoreflex activation in 32 women (OC, *n* = 16; NOC, *n* = 16) performing four trials: 40% handgrip or 80% plantarflexion followed by PECO and arm or leg PM. OC and NOC increased mean arterial pressure (MAP) similarly during handgrip, plantarflexion and arm/leg PECO compared to baseline. Despite increased ventilation (V_E_) during exercise, none of the women exhibited higher V_E_ during arm or leg PECO. OC and NOC similarly increased MAP and V_E_ during arm or leg PM compared to baseline. Therefore, OC and NOC were similar across pressor and ventilatory responses to arm or leg metaboreflex and mechanoreflex activation. However, some differences due to OC may have been masked by disparities in muscle strength. Since women increase V_E_ during exercise, we suggest that while women do not display a ventilatory response to metaboreflex activation (perhaps due to not reaching a theoretical metabolite threshold to stimulate V_E_), the mechanoreflex may drive V_E_ during exercise in women.

## 1 INTRODUCTION

The exercise pressor reflex controls the cardiorespiratory response to dynamic exercise by the joint feedback of the metaboreflex and mechanoreflex, which provides information about the metabolic environment and muscular deformation or tendon stretch of the exercising muscle, respectively. Previous research has demonstrated that women experience a blunted metaboreflex compared to men, yet this is unaffected by the cyclical fluctuations of the menstrual cycle (Assadpour et al., 2020; Jarvis et al., 2011; Lee et al., 2023). Interestingly, Joshi and Edgell (2019) found that using only forearm metaboreflex activation, men increased ventilation whereas women did not (Joshi & Edgell, 2019); other models of upper body metaboreflex activation have not yet investigated sex differences in ventilatory outcomes. Further, using the same forearm occlusion model, Assadpour et al. (2020) observed that women who take oral contraceptives (OC) increased ventilation (V_E_) and tidal volume (Vt) in response to metaboreflex activation via postexercise circulatory occlusion (PECO) after handgrip. While this was the only study to investigate the ventilatory response to metaboreflex activation in OC users, there is conflicting evidence showing either a smaller (Assadpour et al., 2020) or larger increase in mean arterial pressure (MAP) (Minahan et al., 2018; Parmar et al., 2018; Takeda et al., 2021) in OC users during arm PECO compared to naturally cycling women (NOC). The level at which the circulatory occlusion is applied to the arm (i.e., forearm in the former study vs upper arm in the latter studies) could be playing a role in this discrepancy by influencing metabolite concentration. Therefore, one of the primary purposes of the current study is to compare activation of a smaller (forearm) versus a larger (leg) muscle in women to determine if the amount of muscle involved can influence the cardiorespiratory response to single‐limb metaboreflex activation in women while investigating the influence of OC use. Indeed, it has been suggested that differences in the magnitude of the response to activation of the metaboreflex or mechanoreflex could be due to the size and strength of the muscles involved (Lee et al., 2021; Tharpe et al., 2023; Vianna et al., 2010). For example, Lee et al. (2021) and Tharpe et al. (2023) observed that the sex difference in the pressor response to arm metaboreflex activation was attenuated when accounting for strength and muscle mass differences.

Vianna et al. (2010) observed in males that the drop in RR interval induced by passive cycling with four limbs was greater than with a single limb only, suggesting that the muscle mechanoreflex is also dependent on the size or number of the muscles engaged. However, Fouladi et al. (2019) observed that males experienced an increase in blood pressure (BP) in response to arm passive movement (PM; i.e., mechanoreflex activation) but not leg PM, suggesting that limb‐specific differences in mechanoreflex responses are present regardless of muscle size in men. In women, OC users have been shown to have a blunted pressor response compared to NOC during leg PM, yet there was no effect of the menstrual or OC pill cycle on the ventilatory responses to leg PM (Assadpour et al., 2020). The influence of OC on arm PM has not been investigated.

The purpose of the study was to investigate the influence of OC on the cardiorespiratory responses to metaboreflex or mechanoreflex activation in both the forearm and the lower leg while determining if a larger or stronger muscle mass (i.e., leg versus arm) would enhance the cardiorespiratory responses in women. The current study uniquely investigates the influence of OC on the cardiorespiratory responses in the lower limb for metaboreflex activation and in the upper limb for mechanoreflex activation. Since the majority of studies have observed a greater pressor response to arm PECO in OC users, and OC users exhibit exaggerated sympathetic outflow and vascular transduction to arm PECO (D'Souza et al., 2023; Takeda et al., 2021), we hypothesized that OC users would have an augmented pressor response to arm or leg metaboreflex activation compared to NOC. NO‐dependant vasodilation drives the hyperemic response to mechanoreflex activation (Broxterman et al., 2017; Mortensen et al., 2012; Trinity et al., 2012). Given that chronic OC use is associated with increased beta‐receptor sensitivity (Straznicky et al., 1998) and beta‐mediated vasodilation (Limberg et al., 2016), we expect that OC will exhibit a blunted pressor response to PM due to their enhanced ability to vasodilate, as observed in Assadpour et al. (2020). Additionally, OC use may also lead to a hyperventilatory response to forearm or leg PECO due to previous observations that progestin administration has been shown to increase ventilation through an increase in bronchiole smooth muscle relaxation (Foster et al., 1983). Indeed, OC users exhibit an enhanced V_E_ response to forearm PECO compared to NOC (Assadpour et al., 2020); thus, we hypothesized OC would have a hyperventilatory response to both forearm and leg metaboreflex activation compared to NOC. Lastly, we hypothesized that the ventilatory response to either arm or leg PM would be similar in all women due to a lack of previously observed differences in the V_E_ response to leg PM (Assadpour et al., 2020).

## 2 MATERIALS AND METHODS

### 2.1 Ethical approval

This study was conducted in accordance with the ethical and safety standards set by the Declaration of Helsinki, except for database registration. Participants were informed about all experimental protocols and potential risks prior to providing their written consent. The experimental protocols were approved by the Office of Research Ethics at York University (Certificate number: e2018‐254).

### 2.2 Subject characteristics

Healthy individuals were included in this study with no history of any cardiovascular, respiratory, autonomic, or hormonal conditions. Participants were excluded if using medications that may have influenced their cardiorespiratory response or the use of any hormonal contraceptives other than oral contraceptives (OC). Women not taking OC (NOC) were excluded if their menstrual cycle duration was outside of the range of 26–30 days. OC users were required to have been taking their current OC for at least 3 months before testing. All testing occurred during the early follicular (i.e., days 2–5) or placebo pill phase of the menstrual or pill cycle, given the lack of influence of the menstrual or pill cycle on either mechanoreflex or metaboreflex activation (Assadpour et al., 2020). Participants were asked to refrain from consuming fatty foods, alcohol or caffeine, engaging in heavy exercise, and smoking 12 h before their scheduled visit.

Thirty‐two women (OC, *n* = 16 and NOC, *n* = 16) were recruited to participate in the current study. Types of OC included in the current cohort were: Alesse (*n* = 5; Monophasic, 0.1 mg of levonorgestrel, 0.02 mg of ethinyl estradiol (EE)), Alysena (*n* = 3; Monophasic, 0.1 mg of levonorgestrel, 0.02 mg of EE), Yasmin (*n* = 1; Monophasic, 3 mg of dropspirenone, 0.03 mg of EE), Marvelon (*n* = 1; Monophasic, 0.15 mg of desogestrel, 0.03 mg of EE), Linessa (*n* = 1; Triphasic, 0.1, 0.125, and 0.15 mg of desogestrel and 0.025 mg of EE), Ortho Tri‐cyclen (*n* = 1; Triphasic, 0.18, 0.215, and 0.25 mg of norgestimate and 0.035 mg of EE) Ortho Tri‐cyclen Lo (*n* = 1; Triphasic, 0.18, 0.215, and 0.25 mg of norgestimate and 0.025 mg of EE), LoLo (*n* = 1; Biphasic, 0–1 mg norethindrone acetate and 0.01 mg of EE), Freya (*n* = 1; monophasic, 0.15 mg of desogestrel and 0.03 mg of EE) and Aviane (*n* = 1; Monophasic, 0.1 mg of levonorgestrel, and 0.02 mg of EE).

Height and weight were measured using a stadiometer, which was used to calculate body mass index (BMI; height/weight^2^) and body surface area (BSA; 0.007184 × (weight^0.425^) × (height^0.725^)) (Dubois, 1916). Predicted maximal oxygen consumption (VO_2_ max) was estimated using anthropometrics and self‐reported physical activity levels using the Ainsworth equation (Ainsworth, 1992). The thickest and thinnest circumferences of the forearm and calf were measured to estimate muscle volume, as well as the distance between these points. Forearm or calf muscle volume was estimated using a modified equation for the volume of a truncated cone (Podleska et al., 2014).

### 2.3 Experimental protocol

Participants were supine for the duration of data collection, and all exercises and movements were conducted with the limbs on the left side of the body. Prior to participating in any trials, maximum voluntary contraction (MVC) of handgrip and plantarflexion was determined using a pressure transducer and fixed plantarflexion device (MLT004/ST Grip Force, AD Instruments, Colorado Springs, USA), respectively. There were four trials that each participant underwent in a randomized order: upper or lower metaboreflex activation and upper or lower mechanoreflex activation.

### 2.4 Metaboreflex activation

After a 5‐min baseline, participants performed 2 min of isometric exercise maintained at an intensity of 40% MVC for handgrip or 80% MVC for static plantarflexion. The plantarflexion intensity was established during pilot work to achieve equivocal responses to forearm metaboreflex activation. Exercise was followed by 3 min of postexercise circulatory occlusion (PECO) at an occlusion pressure of +50 mmHg above resting systolic blood pressure (SBP) on the exercising limb below the cubital fossa or above the patella, respectively. While traditional arm cuff placement is above the cubital fossa during PECO, the chosen placement was used to maintain consistency with our previously published work that demonstrated an influence of both sex and OC use on the ventilatory responses to forearm metaboreflex activation (Assadpour et al., 2020; Joshi & Edgell, 2019). A 2‐min recovery/reperfusion period followed PECO, and 10 min of rest occurred after all metaboreflex activation trials.

### 2.5 Mechanoreflex activation

Immediately after supine rest (4 min), occlusion was applied distal to the cubital fossa or proximal from the patella for one additional minute prior to PM. Limb occlusion was applied to reduce fluid shifts during limb movement (Venturelli et al., 2017). While occluded, a research assistant passively moved the occluded limb through 90° of flexion to 180° of extension for 3 min, set to the pace of a metronome at 1 Hz or 30 full cycles per minute. A 5‐min resting period followed all mechanoreflex activation trials.

### 2.6 Cardiovascular measures

Heart rate (HR) was measured using a single‐lead electrocardiogram (ECG; BioAMP, ADInstruments, Colorado Springs, USA), and 5 min of continuous ECG was used to analyze resting heart rate variability (HRV) to determine cardiac autonomic balance using the HRV module in the LabChart Pro software (Version 8.1.9, ADInstruments, Colorado, USA). Additionally, HRV was determined using 3 min of continuous ECG during baseline, PECO, and PM for both limbs. BP was determined by continuous beat‐to‐beat finger plethysmography (BMEye Nexfin, Amsterdam, NL) and was calibrated to values from a non‐invasive automated blood pressure monitor (BPTru, VSM MedTech Ltd, Canada). While all tests were conducted while supine, a height corrector was still used to account for BP differences at the heart and finger level. Stroke volume index (SVi) was determined via automated pulse contour analysis (ModelFlow algorithm), which was normalized to body size. Cardiac index (Qi) and total peripheral resistance index (TPRi) were calculated using mean arterial pressure (MAP) and SVi (TPRi = MAP/Qi where Qi = SVi × HR). Hemodynamic averages were calculated from the last 30s of each timepoint: baseline, exercise, PECO, and recovery for metaboreflex activation or baseline and PM for mechanoreflex activation. Blood flow (flow = (π × (diameter/2)^2^) × velocity) was calculated using brachial artery diameter (average of three measures) and blood velocity (1‐min average) captured using duplex ultrasound (Vivid i, GE Healthcare Systems, Canada). Blood velocity was exported to PowerLab using the DAT module (Doppler Audio Translator system, Penn State College of Medicine, Hershey, Pennsylvania). Ultrasound imaging was conducted on the right arm for all trials (i.e., contralateral to the exercising or moving limb), and images were captured within the end of the final minute of each time point.

### 2.7 Ventilatory measures

A heated linear pneumotachometer was used to obtain tidal volume (Vt) and respiratory rate (Series 3813, Hans Rudolph Inc, Shawnee Mission, USA), which were used to calculate V_E_ as a product. Expired gases were measured using carbon dioxide (CO_2_) and oxygen (O_2_) gas analyzers (Model 17630, Vacumed, Ventura, USA).

### 2.8 Data and statistical analysis

All data was acquired at a rate of 1000 Hz through PowerLab (16/35, ADInstruments, Colorado Springs, USA) and LabChart Pro (Version 8.1.9, ADInstruments, Colorado, USA) software. Statistical analyses were performed using Systat SigmaPlot software (Version 15.0, Inpixon, California, USA). Significance was set a priori to *p* ≤ 0.05, and post hoc was conducted using a Bonferroni correction. A one‐way ANOVA was used to determine statistical differences between OC and NOC, such as anthropometrics, estimated fitness, muscular strength, resting BP, and HRV. Some subject characteristics were not normally distributed (i.e., weight, height, and arm volume); therefore, these variables were compared with Kruskal–Wallis ANOVA on Ranks. A two‐way repeated‐measures ANOVA (OC use × Time) was used to determine the cardiorespiratory responses to arm or leg metaboreflex and mechanoreflex activation in both OC and NOC.

As a secondary aim, this study investigated if muscle volume and/or strength influenced the magnitude of the pressor or ventilatory response to arm or leg PECO and PM—considering that these factors are known to influence sex‐related differences in arm metaboreflex activation (Lee et al., 2021; Tharpe et al., 2023). To account for the potential influence of muscle volume and strength, a one‐way ANCOVA was conducted on the change (Δ) in MAP and V_E_ from baseline to either arm or leg PECO or PM during reflex activation in all women, with a post hoc Holm‐Sidak adjustment. Most variables (ΔMAP for arm or leg PECO and PM; ΔV_E_ for leg PECO and arm or leg PM) passed Levene's equal variance test (all *p* > 0.05) and the equal slopes assumption (all *p* > 0.05). During arm PECO, ΔV_E_ did not pass the equal slopes test due to an interaction between the factor (i.e., OC use) and both covariates (i.e., group and handgrip strength, *p* = 0.009; group and forearm muscle volume, *p* = 0.029). Therefore, an Equal Slopes Model of Analysis of Variance was reported for most variables, and individual linear regressions were reported for the ventilatory response in each group during arm PECO. Partial eta^2^ (ηp^2^) was used to estimate effect size. All raw data is displayed as means ± SD, and the adjusted means are displayed as means±SE.

## 3 RESULTS

### 3.1 Subject characteristics and resting heart rate variability (HRV)

Table 1 describes that compared to NOC, OC users were older (*p* = 0.008), had higher SBP (*p* = 0.047), higher diastolic BP (DBP) (*p* = 0.045), and higher MAP (*p* = 0.036). OC also had lower predicted aerobic fitness (*p* = 0.029) and had weaker grip strength than NOC (*p* = 0.048). There were no significant differences between OC and NOC in weight (*p* = 0.27), height (*p* = 0.76), BMI (*p* = 0.25), forearm muscle volume (*p* = 0.29), calf muscle volume (*p* = 0.17), BSA (*p* = 0.27), and maximum plantarflexion force (*p* = 0.89). OC had lower SDRR (*p* = 0.045) and RMSSD (*p* = 0.034) than NOC, but there was no difference in pRR50 (*p* = 0.06), LF power (*p* = 0.08), HF power (*p* = 0.13), and LF/HF ratio (*p* = 0.17).

**TABLE 1:** Subject characteristics (i.e., anthropometrics, resting BP, predicted fitness, muscle strength, and HRV) for OC users, compared to NOC.

	NOC	OC	*p*‐value
Age (years)	21 ± 2	23 ± 3*	**0.008**
Height (cm)	162 ± 7	163 ± 7	0.763
Weight (kg)	60 ± 12	66 ± 13	0.274
Body mass index (kg/m^2^)	23 ± 4	25 ± 4	0.245
Body surface area (m^2^)	1.6 ± 0.2	1.7 ± 0.2	0.271
Systolic blood pressure (mmHg)	102 ± 7	109 ± 10*	**0.047**
Diastolic blood pressure (mmHg)	66 ± 7	72 ± 8*	**0.045**
Mean arterial pressure (mmHg)	78 ± 6	84 ± 8*	**0.036**
Predicted VO_2_ max (mL/kg/min)	40.4 ± 3.0	37.8 ± 3.5*	**0.029**
Estimated Forearm Volume (cm^3^)	0.5 ± 0.1	0.5 ± 0.2	0.290
Estimated Calf Volume (cm^3^)	1.4 ± 0.2	1.6 ± 0.4	0.173
Forearm MVC (N)	288 ± 104	218 ± 75*	**0.048**
Calf MVC (N)	257 ± 143	262 ± 96	0.892
SDRR (ms)	102 ± 41	74 ± 33*	**0.045**
RMSSD (ms)	128 ± 64	82 ± 52*	**0.034**
pRR50 (%)	64 ± 23	49 ± 22	0.059
LF power (nu)	19 ± 11	30 ± 21	0.078
HF power (nu)	79 ± 11	70 ± 21	0.125
LF/HF ratio	0.27 ± 0.23	0.79 ± 1.44	0.167

*Note*: All values are mean ± SD. Significance is represented by bold text.

Abbreviations: HF, high frequency; LF, low frequency; MVC, maximal voluntary contraction; NOC, no oral contraceptive; OC, oral contraceptive; pRR50, proportion of RR interval differences greater than 50 ms; RMSSD, root mean square standard deviation; SDRR, standard deviation of RR intervals; VO_2_ max, maximum oxygen consumption.

* *p* < 0.05.

### 3.2 Arm metaboreflex

**FIGURE 1:**
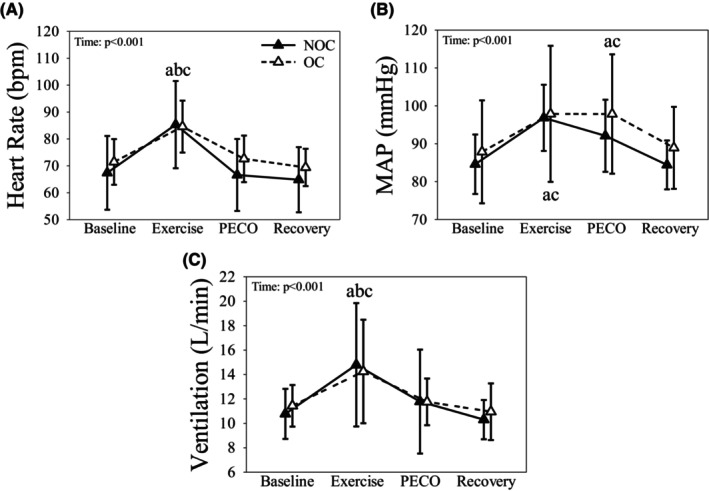
The heart rate (A), mean arterial pressure (MAP; B), and ventilation (C) responses to metaboreflex activation (i.e., postexercise circulatory occlusion; PECO) in the arm of OC (dashed line & open triangles) and NOC females (solid line & closed triangles). Data presented as mean ± SD. a: Indicates significantly different than Baseline in both groups. b: Indicates significantly different than PECO in both groups. c: Indicates significantly different than Recovery in both groups.

In all women, HR was higher during handgrip compared to baseline, arm PECO and recovery (Figure 1A; all *p* < 0.001). During handgrip and arm PECO, MAP was higher than baseline and recovery in both OC and NOC (Figure 1B; all *p* < 0.001). V_E_ was higher during handgrip compared to baseline, arm PECO, and recovery in all women (Figure 1C; all *p* < 0.001).

A main effect of time existed for average brachial artery diameter in the contralateral arm during arm metaboreflex activation (*p* = 0.049; Table 2), yet post hoc analysis revealed no statistical differences (all *p* > 0.10). Brachial blood velocity and flow were higher in OC users than NOC, regardless of time (*p* = 0.026 and *p* = 0.046, respectively), and both variables were higher during handgrip compared to baseline, arm PECO and recovery (all *p* < 0.010; Table 2). Qi and SVi were higher during handgrip than baseline, arm PECO and recovery in OC and NOC (all *p* < 0.001; Table 2). During arm PECO, Qi and SVi were higher than baseline in all women (both *p* < 0.020; Table 2). Additionally, SVi remained elevated during recovery compared to baseline in both groups (*p* = 0.010; Table 2). In all women, TPRi was higher during arm PECO compared to handgrip (*p* = 0.004; Table 2). DBP and SBP were higher during handgrip and arm PECO compared to baseline and recovery (all *p* < 0.001; Table 2).

**TABLE 2:** The influence of OC on the cardiorespiratory response to metaboreflex activation in the arm.

	Arm metaboreflex										
	OC				NOC				*p*‐value		
	Baseline	Exercise	PECO	Recovery	Baseline	Exercise	PECO	Recovery	OC	Time	OC × Time
Brachial artery diameter (cm)	0.31 ± 0.04	0.31 ± 0.04	0.31 ± 0.04	0.30 ± 0.03	0.30 ± 0.03	0.30 ± 0.03	0.29 ± 0.04	0.29 ± 0.04	0.345	**0.049**	0.628
Brachial blood velocity (cm/s)	8.4 ± 3.1^a^	10.7 ± 6.3^a^, ^b^, ^c^, ^d^	8.1 ± 4.1^a^	8.0 ± 3.4^a^	6.1 ± 1.5	6.9 ± 1.6^b^, ^c^, ^d^	5.5 ± 1.5	5.6 ± 1.4	**0.026**	**<0.001**	0.310
Brachial blood flow (L/min/m^2^)	0.61 ± 0.30	0.79 ± 0.49^b^, ^c^, ^d^	0.60 ± 0.37	0.58 ± 0.29	0.44 ± 0.16	0.50 ± 0.15^b^, ^c^, ^d^	0.40 ± 0.19	0.41 ± 0.15	**0.046**	**<0.001**	0.262
Qi (L/min/m^2^)	6.5 ± 0.9	7.5 ± 1.3^b^, ^c^, ^d^	7.0 ± 1.0^b^	6.5 ± 0.9	6.0 ± 1.3	7.0 ± 1.1^b^, ^c^, ^d^	6.2 ± 1.1^b^	6.2 ± 1.2	0.159	**<0.001**	0.132
SVi (mL/m^2^)	91 ± 15	89 ± 14^b^, ^c^, ^d^	98 ± 16^b^	94 ± 17^b^	90 ± 15	84 ± 16^b^, ^c^, ^d^	94 ± 15^b^	96 ± 13 ^b^	0.603	**<0.001**	0.053
TPRi (mmHg/L/min/m^2^)	13.8 ± 2.6	13.3 ± 2.4^c^	14.1 ± 2.2	14.0 ± 2.5	14.7 ± 3.3	14.3 ± 3.0^c^	15.4 ± 3.2	14.2 ± 2.9	0.350	**0.006**	0.198
DBP (mmHg)	75 ± 12	83 ± 15^b^, ^d^	83 ± 13^b^, ^d^	76 ± 9	73 ± 9	83 ± 10^b^, ^d^	79 ± 10^b^, ^d^	72 ± 7	0.572	**<0.001**	0.809
SBP (mmHg)	116 ± 20	124 ± 24^b^, ^d^	127 ± 22^b^, ^d^	118 ± 17	111 ± 8	123 ± 10^b^, ^d^	121 ± 13^b^, ^d^	113 ± 8	0.445	**<0.001**	0.839
Breathing rate (breaths/min)	18 ± 4	21 ± 5^b^, ^c^, ^d^	18 ± 4	17 ± 5	17 ± 3	20 ± 4^b^, ^c^, ^d^	18 ± 3	17 ± 3	0.574	**<0.001**	0.186
ETCO_2_ (mmHg)	41 ± 4	38 ± 5^b^	40 ± 4^b^	41 ± 4	40 ± 3	38 ± 3^b^	38 ± 4^b^	39 ± 2	0.149	**0.002**	0.186
ETO_2_ (mmHg)	113 ± 6	118 ± 11^b^, ^d^	115 ± 6	112 ± 7	114 ± 8	121 ± 9^b^, ^d^	118 ± 11	114 ± 9	0.309	**<0.001**	0.789
Vt (L)	0.65 ± 0.12	0.72 ± 0.41	0.70 ± 0.21	0.70 ± 0.19	0.64 ± 0.16	0.83 ± 0.46	0.66 ± 0.21	0.62 ± 0.11	0.965	0.082	0.441

*Note*: All values are mean ± SD. Significance is represented by bold text (*p* < 0.05).

Abbreviations: DBP, diastolic blood pressure; ETCO_2_, end‐tidal carbon dioxide; ETO_2_, end‐tidal oxygen; NOC, no oral contraceptive; OC, oral contraceptive; PECO, post‐exercise circulatory occlusion; Qi, cardiac index; SBP, systolic blood pressure; SVi, stroke volume index; TPRi, total peripheral resistance index; Vt, tidal volume.

^a^ Indicates significantly different than NOC.

^b^ Indicates significantly different than baseline.

^c^ Indicates significantly different than PECO.

^d^ Indicates significantly different than recovery.

Breathing rate was higher during handgrip compared to baseline, arm PECO, and recovery in all women (all *p* < 0.001; Table 2). End‐tidal CO_2_ (ETCO_2_) was lower during handgrip and arm PECO compared to baseline in all women (both *p* < 0.040; Table 2). In OC and NOC, End‐tidal O_2_ (ETO_2_) was higher during handgrip compared to baseline and recovery (both *p* < 0.005; Table 2). There was no effect of time or OC on Vt during arm metaboreflex activation (all *p* > 0.05; Table 2).

### 3.3 Leg metaboreflex

**FIGURE 2:**
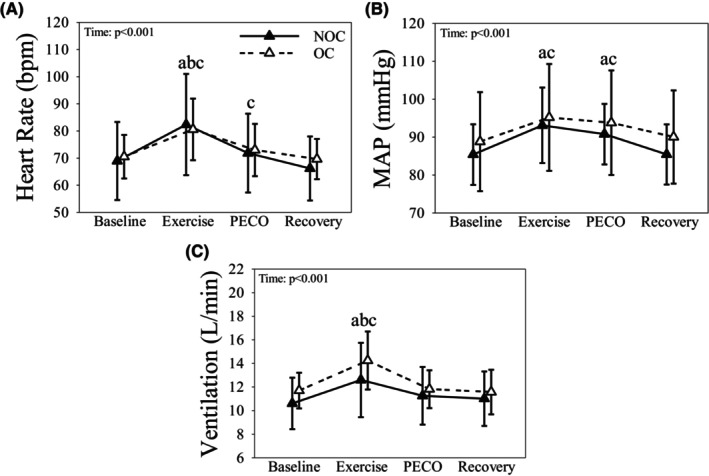
The heart rate (A), mean arterial pressure (MAP; B), and ventilation (C) responses to metaboreflex activation (i.e., postexercise circulatory occlusion; PECO) in the leg of OC (dashed line & open triangles) and NOC females (solid line & closed triangles). Data presented as mean ± SD. a: Indicates significantly different than Baseline in both groups. b: Indicates significantly different than PECO in both groups. c: Indicates significantly different than Recovery in both groups.

HR was higher during plantarflexion compared to baseline, leg PECO and recovery in all women (all *p* < 0.001) and was elevated during leg PECO compared to recovery (*p* = 0.003; Figure 2A). In OC and NOC, MAP was higher during plantarflexion and leg PECO compared to baseline and recovery (all *p* < 0.001; Figure 2B). V_E_ was higher during plantarflexion compared to baseline, leg PECO and recovery in all women (all *p* < 0.001; Figure 2C).

During leg metaboreflex activation, there was no effect of OC use or time on the average contralateral brachial artery diameter (all *p* > 0.10). OC had higher brachial blood velocity and flow than NOC, regardless of time (*p* < 0.030; Table 3). There was also a main effect of time for brachial blood velocity (*p* = 0.024); however, post hoc analysis revealed that there were no differences between any time points (*p* > 0.05). In all women, Qi was higher during plantarflexion compared to baseline, leg PECO and recovery (all *p* < 0.001), and Qi was elevated during leg PECO compared to baseline and recovery in OC and NOC (both *p* < 0.005; Table 3). In all women, SVi and TPRi were lower during plantarflexion compared to baseline, leg PECO and recovery (all *p* < 0.035; Table 3), and DBP and SBP were higher during plantarflexion and leg PECO compared to baseline and recovery (all *p* < 0.001; Table 3). Additionally, DBP was also higher during plantarflexion compared to leg PECO (*p* = 0.044; Table 3).

**TABLE 3:** The influence of OC on the cardiorespiratory response to metaboreflex activation in the leg.

	Leg metaboreflex										
	OC				NOC				*p*‐value		
	Baseline	Exercise	PECO	Recovery	Baseline	Exercise	PECO	Recovery	OC	Time	OC × Time
Brachial artery diameter (cm)	0.31 ± 0.04	0.31 ± 0.03	0.31 ± 0.03	0.32 ± 0.03	0.29 ± 0.04	0.30 ± 0.04	0.30 ± 0.04	0.30 ± 0.04	0.252	0.197	0.612
Brachial blood velocity (cm/s)	8.3 ± 3.0^a^	9.0 ± 4.4^a^	9.6 ± 5.2^a^	8.3 ± 3.8^a^	6.1 ± 1.4	6.7 ± 2.2	6.1 ± 1.9	5.7 ± 1.3	**0.020**	**0.024**	0.188
Brachial blood flow (L/min/m^2^)	0.63 ± 0.27^a^	0.69 ± 0.39^a^	0.75 ± 0.48^a^	0.65 ± 0.35^a^	0.42 ± 0.15	0.46 ± 0.17	0.46 ± 0.21	0.42 ± 0.16	**0.027**	0.054	0.375
Qi (L/min/m^2^)	6.6 ± 0.7	7.3 ± 0.7^b^, ^c^, ^d^	6.9 ± 0.7^b^, ^d^	6.7 ± 0.7	6.3 ± 1.1	7.2 ± 1.1^b^, ^c^, ^d^	6.6 ± 1.1^b^, ^d^	6.2 ± 0.9	0.295	**<0.001**	0.336
SVi (mL/m^2^)	94 ± 13	93 ± 13^c^, ^d^	96 ± 12	97 ± 13	92 ± 10	89 ± 12^c^, ^d^	94 ± 10	95 ± 10	0.540	**<0.001**	0.664
TPRi (mmHg/L/min/m^2^)	13.5 ± 1.9	13.0 ± 1.5^b^, ^c^, ^d^	13.6 ± 1.8	13.6 ± 1.9	14.0 ± 2.9	13.2 ± 2.6^b^, ^c^, ^d^	14.0 ± 2.6	14.1 ± 2.6	0.628	**<0.001**	0.867
DBP (mmHg)	76 ± 11	81 ± 12^b^, ^c^, ^d^	80 ± 11^b^, ^d^	77 ± 10	73 ± 9	80 ± 10^b^, ^c^, ^d^	77 ± 8^b^, ^d^	73 ± 8	0.430	**<0.001**	0.273
SBP (mmHg)	119 ± 17	124 ± 19^b^, ^d^	124 ± 18^b^, ^d^	120 ± 17	113 ± 11	120 ± 11^b^, ^d^	120 ± 10^b^, ^d^	114 ± 11	0.306	**<0.001**	0.447
Breathing rate (breaths/min)	18 ± 4	21 ± 6^b^, ^c^, ^d^	18 ± 4	18 ± 4	19 ± 4	22 ± 8^b^, ^c^, ^d^	19 ± 4	19 ± 3	0.671	**<0.001**	0.981
ETCO_2_ (mmHg)	41 ± 4	40 ± 4	40 ± 3^b^	41 ± 3	39 ± 2	39 ± 2	38 ± 3^b^	39 ± 2	0.105	**0.015**	0.194
ETO_2_ (mmHg)	113 ± 6	117 ± 5^b^	115 ± 6^b^	114 ± 6	115 ± 7	117 ± 7^b^	118 ± 7^b^	117 ± 6	0.346	**<0.001**	0.202
Vt (L)	0.68 ± 0.21	0.73 ± 0.27	0.68 ± 0.18	0.67 ± 0.13	0.59 ± 0.19	0.62 ± 0.22	0.63 ± 0.19	0.60 ± 0.16	0.191	0.369	0.733

*Note*: All values are mean ± SD. Significance is represented by bold text (*p* < 0.05).

Abbreviations: DBP, diastolic blood pressure; ETCO_2_, end‐tidal carbon dioxide; ETO_2_, end‐tidal oxygen; NOC, non‐oral contraceptive; OC, oral contraceptive; PECO, post‐exercise circulatory occlusion; Qi, cardiac index; SBP, systolic blood pressure; SVi, stroke volume index; TPRi, total peripheral resistance index; Vt, tidal volume.

^a^ Indicates significantly different than NOC.

^b^ Indicates significantly different than baseline.

^c^ Indicates significantly different than PECO.

^d^ Indicates significantly different than recovery.

In all women, breathing rate was higher during plantarflexion compared to baseline, leg PECO, and recovery (all *p* < 0.005; Table 3). ETCO_2_ was lower during leg PECO compared to baseline (*p* = 0.013), while ETO_2_ was higher during plantarflexion and leg PECO compared to baseline in both OC and NOC (both *p* < 0.005; Table 3). During leg metaboreflex, there was no main effect of time or OC use nor any interaction for Vt in OC and NOC (all *p* > 0.10; Table 3).

### 3.4 Arm mechanoreflex

**FIGURE 3:**
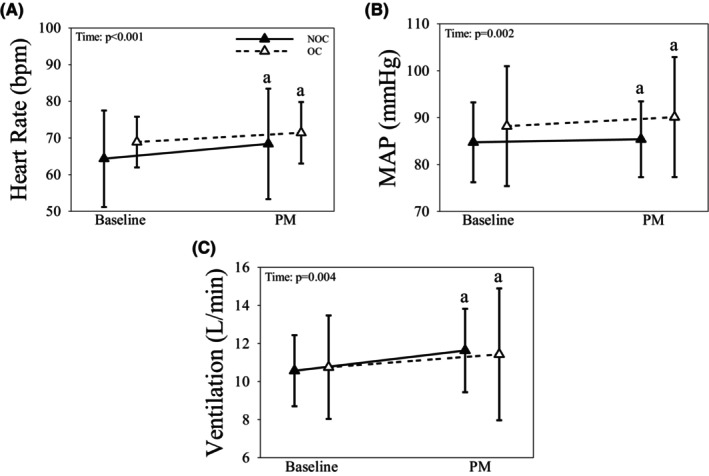
The heart rate (A), mean arterial pressure (MAP; B), and ventilation (C) responses to mechanoreflex activation (i.e., passive movement; PM) in the arm in OC (dashed line & open triangles) and NOC (solid line & closed triangles) females. Data presented as mean ± SD. a: Indicates significantly different than Baseline within group.

In both groups, HR, MAP, and V_E_ increased during arm PM compared to baseline (all *p* < 0.005; Figure 3A–C). OC users had higher brachial blood velocity and flow compared to NOC, regardless of time (both *p* < 0.030; Table 4). In response to arm PM, both contralateral brachial artery diameter and TPRi decreased from baseline (both *p* < 0.040), and Qi, DBP and SBP increased from baseline (all *p* < 0.005; Table 4) in all women. SVi was unaffected by time or OC use (all *p* > 0.10; Table 4). In OC and NOC, breathing rate and ETO_2_ increased (both *p* < 0.010), while ETCO_2_ decreased during arm PM compared to baseline (*p* = 0.004; Table 4). There was no effect of time or OC use on Vt response to arm PM (all *p* > 0.55; Table 4).

**TABLE 4:** The influence of OC on the cardiorespiratory response to arm PM.

	Arm mechanoreflex						
	OC		NOC		*p*‐value		
	Baseline	PM	Baseline	PM	OC	Time	OC × Time
Brachial artery diameter (cm)	0.31 ± 0.04	0.31 ± 0.04^b^	0.30 ± 0.04	0.29 ± 0.03^b^	0.406	**0.016**	0.453
Brachial blood velocity (cm/s)	8.7 ± 3.9^a^	8.6 ± 3.7^a^	5.7 ± 2.1	5.7 ± 1.9	**0.012**	0.783	0.842
Brachial blood flow (L/min/m^2^)	0.63 ± 0.34^a^	0.62 ± 0.31^a^	0.41 ± 0.17	0.40 ± 0.16	**0.024**	0.397	0.974
Qi (L/min/m^2^)	6.5 ± 0.6	6.7 ± 0.7^b^	5.9 ± 1.5	6.2 ± 1.5^b^	0.243	**0.001**	0.755
SVi (mL/m^2^)	95 ± 14	95 ± 14	92 ± 15	91 ± 14	0.524	0.135	0.235
TPRi (mmHg/L/min/m^2^)	13.8 ± 2.6	13.7 ± 2.3^b^	15.2 ± 4.4	14.7 ± 4.1^b^	0.333	**0.038**	0.226
DBP (mmHg)	75 ± 11	77 ± 11^b^	73 ± 9	74 ± 8^b^	0.424	**<0.001**	0.133
SBP (mmHg)	117 ± 16	120 ± 17^b^	112 ± 10	113 ± 11^b^	0.212	**0.002**	0.101
Breathing rate (breaths/min)	19 ± 3	21 ± 4^b^	19 ± 2	21 ± 4^b^	0.892	**0.002**	0.533
ETCO_2_ (mmHg)	41 ± 4	40 ± 4^b^	39 ± 3	39 ± 2^b^	0.127	**0.004**	0.751
ETO_2_ (mmHg)	112 ± 6	115 ± 5^b^	115 ± 7	116 ± 7^b^	0.338	**0.009**	0.580
Vt (L)	0.59 ± 0.17	0.58 ± 0.19	0.57 ± 0.10	0.57 ± 0.16	0.826	0.562	0.665

*Note*: All values are mean ± SD. Significance is represented by bold text (*p* < 0.05).

Abbreviations: DBP, diastolic blood pressure; ETCO_2_, end‐tidal carbon dioxide; ETO_2_, end‐tidal oxygen; NOC, non‐oral contraceptive; OC, oral contraceptive; PM, passive movement; Qi, cardiac index; SBP, systolic blood pressure; SVi, stroke volume index; TPRi, total peripheral resistance index; Vt, tidal volume.

^a^ Indicates significantly different than NOC.

^b^ Indicates significantly different than baseline.

### 3.5 Leg mechanoreflex

**FIGURE 4:**
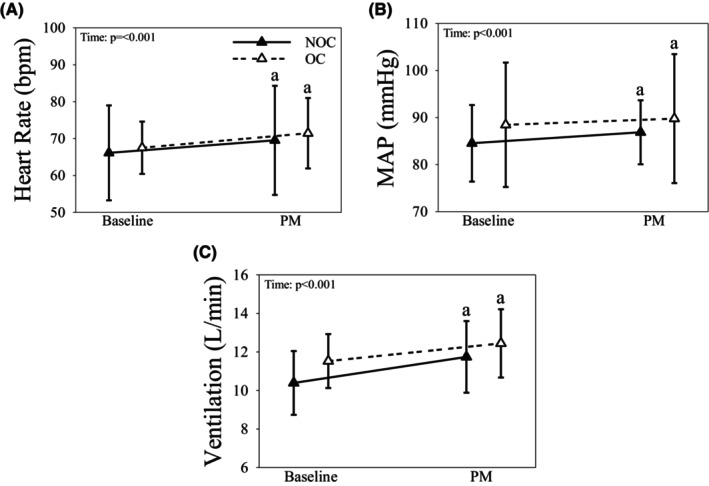
The heart rate (A), mean arterial pressure (MAP; B), and ventilation (C) responses to mechanoreflex activation (i.e., passive movement; PM) in the leg in OC (dashed line & open triangles) and NOC (solid line & closed triangles). Data presented as mean ± SD. a: Indicates significantly different than Baseline within group.

HR (Figure 4A), MAP (Figure 4B), and V_E_ (Figure 4C) increased during leg PM in all women (all *p* < 0.001). None of mean brachial artery diameter, blood velocity, blood flow, nor SVi were affected by OC use or time during leg mechanoreflex activation (all *p* > 0.10; Table 5). Qi, DBP, and SBP increased from baseline during leg PM (all *p* < 0.006; Table 5), yet TPRi decreased in both OC and NOC (*p* = 0.019; Table 5). In NOC only, breathing rate increased from baseline (*p* < 0.001; Table 5), and ETCO_2_ decreased from baseline during leg PM in OC and NOC (*p* < 0.001; Table 5). Vt and ETO_2_ were unaffected by time or OC use (all *p* > 0.10; Table 5).

**TABLE 5:** The influence of OC on the cardiorespiratory response to leg PM.

	Leg mechanoreflex						
	OC		NOC		*p*‐value		
	Baseline	PM	Baseline	PM	OC	Time	OC × Time
Brachial artery diameter (cm)	0.31 ± 0.03	0.31 ± 0.03	0.30 ± 0.04	0.30 ± 0.04	0.550	0.629	0.764
Brachial blood velocity (cm/s)	7.0 ± 2.9	7.7 ± 3.6	5.8 ± 2.0	5.9 ± 2.1	0.137	0.120	0.198
Brachial blood flow (L/min/m^2^)	0.52 ± 0.25	0.57 ± 0.32	0.41 ± 0.18	0.42 ± 0.17	0.164	0.109	0.184
Qi (L/min/m^2^)	6.3 ± 0.7	6.7 ± 0.8^a^	6.0 ± 1.0	6.2 ± 1.1^a^	0.209	**<0.001**	0.506
SVi (mL/m^2^)	95 ± 15	95 ± 14	91 ± 11	91 ± 10	0.361	0.847	0.739
TPRi (mmHg/L/min/m^2^)	14.1 ± 2.3	13.5 ± 2.4^a^	14.6 ± 3.5	14.4 ± 3.4^a^	0.500	**0.019**	0.277
DBP (mmHg)	75 ± 11	76 ± 11^a^	72 ± 8	75 ± 7^a^	0.497	**<0.001**	0.188
SBP (mmHg)	118 ± 20	119 ± 21^a^	112 ± 9	115 ± 8^a^	0.397	**0.005**	0.306
Breathing rate (breaths/min)	19 ± 3	20 ± 4	18 ± 4	21 ± 4^a^	0.934	**<0.001**	**0.016**
ETCO_2_ (mmHg)	41 ± 3	40 ± 3^a^	39 ± 2	38 ± 2^a^	0.111	**<0.001**	0.845
ETO_2_ (mmHg)	116 ± 12	116 ± 5	115 ± 6	118 ± 6	0.851	0.235	0.229
Vt (L)	0.62 ± 0.07	0.65 ± 0.16	0.58 ± 0.12	0.57 ± 0.11	0.172	0.545	0.136

*Note*: All values are mean ± SD. Significance is represented by bold text (*p* < 0.05).

Abbreviations: DBP, diastolic blood pressure; ETCO_2_, end‐tidal carbon dioxide; ETO_2_, end‐tidal oxygen; NOC, non‐oral contraceptive; OC, oral contraceptive; PM, passive movement; Qi, cardiac index; SBP, systolic blood pressure; SVi, stroke volume index; TPRi, total peripheral resistance index; Vt, tidal volume.

^a^ Indicates significantly different than baseline.

### 3.6 Heart rate variability during PECO and PM

During arm metaboreflex activation, SDRR increased in response to arm PECO in both OC and NOC (*p* = 0.021), yet there was no effect of time or group on RMSSD, pRR50, LF power, HF power, and LF/HF ratio (all *p* > 0.05; Table S1). Compared to NOC, SDRR tended to be lower in OC during leg metaboreflex activation irrespective of time (*p* = 0.052; Table S1). In all women, during leg PECO, RMSSD and pRR50 decreased (*p* = 0.024 and *p* = 0.002, respectively), whereas HF power, LF power and LF/HF ratio were unaffected by OC or leg PECO (all *p* > 0.10; Table S1).

Regardless of time, SDRR was significantly lower (*p* = 0.040), and RMSSD tended to be lower in OC compared to NOC during arm mechanoreflex activation (*p* = 0.052; Table S2). SDRR, RMSSD, and pRR50 all decreased during arm PM compared to baseline in OC and NOC (all *p* < 0.030), yet neither OC use nor arm PM affected HF power, LF power, or LF/HF ratio (all *p* > 0.09; Table S2). During leg PM, SDRR, RMSSD, pRR50, and HF power decreased in both OC and NOC (all *p* < 0.005; Table S2). In contrast, LF power increased during leg mechanoreflex activation in all women (*p* = 0.003; Table S2). There was no effect of leg PM or OC use for the LF/HF ratio (all *p* > 0.10; Table S2).

### 3.7 Influence of muscle strength and size

During arm PECO, the ΔV_E_ failed the equal slopes assumption; thus, linear regressions were calculated in individual groups. In NOC, neither arm volume (R^2^ = 0.243, F (1, 12) = 3.849, *p* = 0.07) nor handgrip strength (R^2^ = 0.252, F (1, 13) = 4.384, *p* = 0.06) influenced ΔV_E_ during arm metaboreflex activation. During arm PECO in OC, neither arm volume (R^2^ = 0.002, F (1, 13) = 0.022, *p* = 0.88) nor handgrip strength (R^2^ = 0.008, F (1, 14) = 0.117, *p* = 0.734) influenced ΔV_E_. The ANCOVA model, which adjusted for muscle volume or strength, was able to account for 12–41% of the variability (R^2^) in the pressor responses to arm or leg metaboreflex and mechanoreflex activation; however, the model was less able to account for the variability (3%–26%) for the ventilatory response to leg PECO and arm or leg PM (Table S3). Handgrip strength significantly influenced ΔMAP arm PECO response (*p* < 0.001; large effect *ηp* ^2^ > 0.14; Table S3). After adjustment, OC had an enhanced pressor response to arm PECO than NOC (*p* = 0.038; large effect ηp^2^ > 0.14; Table S3). During leg metaboreflex activation, plantarflexion strength significantly influenced ΔMAP to leg PECO (*p* = 0.006; large effect ηp^2^ > 0.14), yet there still were no differences between OC and NOC post‐adjustment (*p* > 0.1; Table S3). During leg PM, plantarflexion strength significantly influenced ΔV_E_ (*p* = 0.045; large effect *ηp* ^2^ > 0.14); however, this did not translate into any differences between either group after adjusting for the covariates (*p* > 0.9; Table S3). There was no effect of the main factor of OC use, nor an effect of the covariates muscle strength or volume on ΔMAP during arm or leg PM (all *p* > 0.1) or ΔV_E_ during leg PECO or arm PM (all *p* > 0.2; Table S3).

## 4 DISCUSSION

The cardiorespiratory responses to exercise during metaboreflex or mechanoreflex activation were similar when comparing OC and NOC. Interestingly, neither group increased V_E_ during arm or leg PECO while exhibiting similar pressor responses. Considering that exercise and PM increases V_E_, the above suggests that women may more heavily rely on afferent signaling from mechanoreflex activation during exercise to drive V_E_, which may be due to some unmet theoretical metabolite threshold necessary to stimulate ventilation or sexually dimorphic reflex control. Lastly, covariate analysis demonstrated that muscle strength may have masked some differences between OC and NOC in the pressor response during arm or leg PECO but not arm or leg PM.

Contrary to our hypothesis, we did not observe any differences between OC and NOC in the ventilatory or pressor responses to metaboreflex activation. Previously, Joshi and Edgell (2019) observed that women do not increase V_E_ during arm PECO compared to men, which the authors postulated was potentially due to women having smaller muscles leading to reduced metabolite accumulation. Considering that the current study also investigated the ventilatory response to metaboreflex activation in the leg (i.e., a larger muscle mass) and we did not observe an increased V_E_ during leg PECO, this may suggest that all women may exhibit a blunted respiratory response to metaboreflex activation, regardless of OC use. Further, since it is well‐established that women increase V_E_ during exercise, the mechanoreflex may be primarily responsible for providing sufficient input to stimulate V_E_ in women. Previous research observed that OC users had an increased ventilatory response to arm metaboreflex activation (Assadpour et al., 2020), yet we did not observe that in the current study. Unpublished data from Assadpour et al. (2020) suggests that their cohort was evenly matched for forearm strength (OC: 216 ± 59 N vs. NOC: 221 ± 58 N; *p* = 0.9), whereas our NOC group was stronger in the current study; therefore, any potential influences of OC on the ventilatory response to metaboreflex activation may have been confounded by disparities in muscle strength. In the present study, the covariate analysis demonstrated that handgrip strength exerted some influence on the variation in the ventilatory response to metaboreflex activation.

Unexpectedly, OC did not display a greater pressor response to metaboreflex activation, as observed in previous literature (Minahan et al., 2018; Parmar et al., 2018; Takeda et al., 2021). Cuff placement may have been responsible for this disparity, as the aforementioned studies used upper arm PECO rather than forearm PECO, which could have potentially captured a greater number of metabolites and, thus, increased the pressor response to a greater degree. Since the NOC group was stronger than OC in the current study, NOC could have potentially generated a greater concentration of metabolites, confounding our results. Previous research comparing sexes suggests that the pressor response to arm metaboreflex activation is related to muscle strength (Lee et al., 2021; Tharpe et al., 2023) or muscle size (Tharpe et al., 2023). Indeed, OC users had a greater pressor response to PECO in the arm after adjusting for muscular strength (Table S3)—yet a lack of difference between OC and NOC in V_E_ remained after controlling for these factors.

Our observation that women do not increase ventilation during metaboreflex activation is intriguing. There have been multiple studies showing interactions between the chemoreflexes and the metaboreflex (Alghaith et al., 2019; Boulet et al., 2022; de Oliveira et al., 2023; Delliaux et al., 2015; Edgell & Stickland, 2014; Houssiere et al., 2005; Houssière et al., 2006; Lykidis et al., 2009, 2010; Wan et al., 2020). Of those studies, most researchers investigated the reflex interactions in men only (Alghaith et al., 2019; Delliaux et al., 2015; Edgell & Stickland, 2014; Houssière et al., 2006) or in mixed‐sex groups (de Oliveira et al., 2023; Houssiere et al., 2005; Lykidis et al., 2009, 2010; Wan et al., 2020). There was only a single study that investigated sex differences (Boulet et al., 2022), which observed no sex differences in the ventilatory response to hypoxic PECO; however, neither menstrual cycle nor hormonal contraceptives were controlled for in the female group. It was previously observed that the CO_2_ chemoreflex does not differ between OC and NOC (Assadpour et al., 2020), yet to our knowledge, it is unknown if OC use influences the hypoxic chemoreflex. Van Klaveren and Demedts (1998) suggest that individuals with similar lung size should have similar V_E_ responses to hypoxia; therefore, we hypothesize that OC use will not influence the peripheral chemoreflex. Further, we suggest that there is no influence of OC use on the interactions between the metaboreflex and chemoreflexes since both groups in the current study had similar changes in ETCO_2_ and ETO_2_ with similar cardiovascular responses during PECO.

Our lab previously observed that OC users have reduced blood pressure responses to leg PM compared to NOC (Assadpour et al., 2020). Considering that Limberg et al. (2016) observed an enhanced nitric oxide (NO)‐mediated vasodilatory capacity in OC compared to NOC and that the local hyperaemic response to leg PM is NO‐dependant (Mortensen et al., 2012), this could contribute to the previously observed reduced pressor response to leg PM in OC compared to NOC. In the current study, there were no differences between OC and NOC in the absolute pressor responses during arm or leg PM. Covariate analysis demonstrated that neither leg volume nor strength influenced the pressor responses to arm or leg PM in the current study; however, differences in OC formulations could have contributed to the lack of observed differences. The current study included some participants who used lower hormonal dosage OC compared to Assadpour et al. (2020), which may have exposed the current OC users to a smaller estrogenic dosage. Administering EE alone increases endothelium‐dependant vasodilation in young, healthy women (Meendering et al., 2009); although, specific progestin types, such as levonorgestrel (Thompson et al., 2011) and desogestrel (Meendering et al., 2009), in combined OC antagonizes these vasodilatory effects. Both studies included similar numbers of progestin types, yet the current study included lower doses of estrogen and progesterone; therefore, the lack of observed differences in the pressor response to PM may be due to less exogenous hormone exposure.

As our primary index of sympathetic outflow, we measured brachial artery diameter and flow of the non‐exercising arm throughout each protocol. Interestingly, we only noted a slight reduction of diameter during arm PM, potentially indicating increased sympathetic outflow. However, this did not translate to a change in flow. We observed increased brachial flow during arm exercise, likely a result of greater driving pressure, yet this was not observed during leg exercise or limb PM. Previous observations have shown that after infusion with a NO synthase inhibitor, the hyperemic response of the inactive upper limb was significantly diminished at low‐moderate intensities (60 & 80 watts) of leg cycling; however, this non‐active hyperemic response remained intact during contralateral handgrip exercise (Green et al., 2005). Additionally, Thijssen et al. (2009) demonstrated that varying modalities of lower limb exercise (i.e., cycling, leg kicking, and walking) induced altered patterns of blood flow in the non‐active upper limb. Taken together, this may suggest that lower limb plantarflexion may not have been a sufficient shear stress to increase contralateral non‐active brachial flow.

At odds with our initial hypothesis, OC users did not display a greater pressor response, nor did their brachial diameters reflect changes in sympathetic vascular transduction. There may be a balance between enhanced vasodilatory capacity (Limberg et al., 2016) and the hypertensive effects of OC (Zuhaira et al., 2022) that contribute to the lack of observed differences. However, OC users had higher brachial velocity and flow compared to NOC at all time points, potentially indicating a predominance of the vasodilatory effect of OC or anatomical differences. Alternatively, it is theoretically possible that the increased brachial blood flow observed in OC users could be due to a greater proportion of type I skeletal muscle fibers, which can create more vasoactive metabolites and could be evidenced by the lower strength of the OC women.

The parasympathetic influence (i.e., SDRR) on cardiac control increased during arm PECO in both groups, yet the opposite was true for leg PECO, where parasympathetic cardiac control (i.e., RMSSD and pRR50) decreased during leg PECO in all women. A concurrent lack of change in LF/HF (i.e., an index of cardiac sympathovagal balance) indices may indicate that the cardiac changes to isolated metaboreflex activation are mitigated by parasympathetic withdrawal in women. In support of this, previous research has also demonstrated that HRV increases during arm PECO, even when controlling for tidal ventilation and breathing rate (Nishiyasu et al., 1994). We suggest that PECO of a smaller muscle mass (i.e., arm) may activate both cardiac vagal activity (Kluess & Wood, 2005) and muscle sympathetic nerve activity (MSNA) (Kamiya et al., 2001), but this cardiac vagal activity may be suppressed in the face of a larger muscle mass (i.e., leg) (Fisher et al., 2013) and potentially greater MSNA (Doherty et al., 2019). In all women, parasympathetic cardiac control decreased during PM in both limbs (i.e., SDRR, RMSSD, and pRR50); however, LF power (i.e., an index of combined sympathetic and parasympathetic influence on HR (Akselrod et al., 1981), or baroreflex sensitivity (Goldstein et al., 2011)) only increased during leg PM. Vianna et al. (2010) observed a greater reduction in RR interval when more limbs or a larger muscle mass was involved in passive cycling. Larger muscle volume may mediate an increase in cardiac sympathetic control during leg PM in both OC and NOC, while smaller muscles (i.e., arm) may rely more on cardiac parasympathetic withdrawal.

### 4.1 Limitations

The forearm cuff placement used for metaboreflex activation deviates from the classical model, which may have resulted in a smaller accumulation of metabolites compared to previous studies in the literature—considering that a smaller muscle volume was occluded. However, the *a priori* purpose of this study was to duplicate our own previous findings using this model (i.e., that women do not increase V_E_ during forearm PECO (Joshi & Edgell, 2019)) while concurrently investigating the cardiorespiratory response in a larger muscle mass (i.e., the leg) to determine if a greater metabolite accumulation would lead to greater cardiorespiratory responses. Additionally, circulatory occlusion was applied to reduce fluid shifts during limb PM, which may have generated some metabolites. Importantly, we did not observe a ventilatory response to PECO (i.e., a more potent stimulus of metabolite generation), yet we did observe an increased ventilatory response to PM; thus, it is unlikely that metabolite accumulation during PM influenced ventilatory responses.

It is important to note that a sympathetic impulse requires the transduction of that impulse by the vasculature (i.e., neurovascular transduction) to change hemodynamics; therefore, using brachial velocity and diameter of the non‐exercising limb as a marker of sympathetic outflow may not entirely represent the extent of sympathetic outflow particularly in women. Compared to men, premenopausal women exhibit a non‐significant relationship between MSNA and TPR, which is suggestive of a diminished capacity to transduce sympathetic impulses (Hart et al., 2009). This dampening of sympathetic outflow translating into vasoconstriction may be due to the fact that women have greater β‐mediated vasodilation counteracting α‐adrenergic vasoconstriction (Hart et al., 2011; Kneale et al., 2000). Additionally, OC users have greater β‐mediated vasodilation during the placebo pill phase than NOC in the early follicular phase (Limberg et al., 2016). Yet, D'Souza et al. (2023) observed greater sympathetic vascular transduction in OC users compared to NOC in response to static handgrip. Therefore, differences in neurovascular transduction rather than sympathetic output *per se* may contribute to changes in brachial velocity or diameter in response to exercise or PM in the current study.

While the type (mono vs. triphasic), hormonal dosage, and generation of OC (i.e., progestin type) were recorded, they were not controlled for; however, at least half of OC users were on a similar monophasic OC with identical progestin and EE doses (i.e., Alesse and Alysena). There is limited data investigating the effects of varying doses over the pill cycle (mono vs. bi or triphasic OC) on autonomic or respiratory function; however, Harvey and colleagues (Harvey et al., 2015) did not observe any differences between mono, bi, or triphasic OC in resting MAP or muscle sympathetic nervous activity. Previous research has demonstrated that second‐generation OC reduces endothelial function as observed via decreased flow‐mediated dilation (FMD) (Franceschini et al., 2013; Heidarzadeh et al., 2014; Lizarelli et al., 2009), yet a fourth‐generation OC had no influence on FMD (Giribela et al., 2012). Given that the FMD response is at least partly mediated by NO (Green et al., 2014), this may suggest that varying OC generations may have different effects on NO‐dependant vasodilation. This could influence the pressor response and may have contributed to some of the variation in the current cohort of OC users. Indeed, OC users had almost double the standard deviation in MAP at each timepoint during arm metaboreflex activation and both arm and leg mechanoreflex activation. Future research should consider comparing the pressor response to metaboreflex or mechanoreflex activation across pill generations or more tightly controlling for a single type of OC.

It is also important to note that other routes of synthetic hormone administration, such as patches, implants, or intrauterine devices, may have divergent physiological responses to exercise pressor reflex activation or effects on vascular function; thus, this study cannot be extended to other hormonal contraceptives. Furthermore, this study was conducted in relatively healthy young women and should not be extended to older, obese, or diseased populations, who may have additional influences exacerbating autonomic dysfunction.

## 5 CONCLUSIONS

In conclusion, there was no influence of OC use on the cardiovascular or ventilatory response to upper or lower body PECO or PM. The lack of observed differences between OC and NOC may be due to potential differences in strength, considering that OC users had weaker forearms and most previous research was conducted in cohorts that did not differ in strength. Neither OC nor NOC exhibited an increased ventilatory response during arm or leg metaboreflex activation—suggesting that metaboreflex activation may not stimulate breathing in women or that women may not meet a theoretical metabolite accumulation threshold necessary to stimulate V_E_. All women had similar increased pressor and ventilatory responses to arm and leg mechanoreflex activation, suggesting that there are no limb‐specific differences. Taken together, our results suggest that the mechanoreflex may be primarily responsible for initiating a ventilatory response in women during exercise or that concurrently activating the mechanoreflex provides additional input required to increase ventilation during dynamic exercise.

## AUTHOR CONTRIBUTIONS

The experimental protocols were performed in the Women's Cardiovascular Health lab at York University. Both authors (TJP and HE) were responsible for the study design, ethics application, participant recruitment, data collection, data analysis, manuscript preparation and revisions. All authors have approved the final version of this manuscript for publication.

## FUNDING INFORMATION

This project was partly funded by the Natural Sciences and Engineering Research Council of Canada and an Ontario Graduate Scholarship awarded to TJP.

## CONFLICT OF INTEREST STATEMENT

The authors have no competing interests to declare.

## Supporting information


Table S1‐3.

## Data Availability

This is not applicable to the current manuscript.
