# Supplementary material for: The influence of oral contraceptives on the exercise pressor reflex in the upper and lower body
Source: Physiol Rep. 2024 Jul 11;12(13):e16144. doi: 10.14814/phy2.16144 (PMC11239320; doi:10.14814/phy2.16144)
Supplement: Supplementary file 1 — Table S1‐3. [file PHY2-12-e16144-s001.docx]

**Supplemental Information**

**Supplemental Table 1.** The influence of OC on the HRV response to arm or leg metaboreflex activation.

|  | PECO | | | | | | | | | | | | | | |
| --- | --- | --- | --- | --- | --- | --- | --- | --- | --- | --- | --- | --- | --- | --- | --- |
|  | Arm | | | | | | |  | Leg | | | | | | |
|  | OC | | NOC | | P-value | | |  | OC | | NOC | | P-value | | |
|  | Baseline | PECO | Baseline | PECO | OC | Time | OC  X Time |  | Baseline | PECO | Baseline | PECO | OC | Time | OC  X Time |
| SDRR (ms) | 71±40 | 83±45 ^a^ | 99±42 | 113±53 ^a^ | 0.070 | **0.021** | 0.823 |  | 72±42 | 63±35 | 95±26 | 89±40 | 0.052 | 0.133 | 0.801 |
| RMSSD (ms) | 83±61 | 90±72 | 126±61 | 271±569 | 0.157 | 0.270 | 0.314 |  | 83±60 | 69±62 ^a^ | 119±46 | 104±66 ^a^ | 0.085 | **0.024** | 0.965 |
| pRR50 (%) | 49±26 | 44±30 | 61±22 | 59±23 | 0.136 | 0.199 | 0.462 |  | 49±20 | 37±30 ^a^ | 62±22 | 52±24 ^a^ | 0.088 | **0.002** | 0.665 |
| LF (nu) | 32±24 | 36±25 | 19±10 | 25±17 | 0.061 | 0.191 | 0.925 |  | 27±20 | 31±19 | 22±14 | 22±11 | 0.159 | 0.479 | 0.557 |
| HF (nu) | 67±22 | 62±24 | 77±9 | 73±16 | 0.088 | 0.180 | 0.942 |  | 72±19 | 68±18 | 75±13 | 75±11 | 0.251 | 0.466 | 0.568 |
| LF/HF ratio | 0.97±1.97 | 1.00±1.29 | 0.27±0.17 | 0.42±0.44 | 0.060 | 0.763 | 0.824 |  | 0.86±2.14 | 0.57±0.47 | 0.33±0.30 | 0.32±0.21 | 0.215 | 0.540 | 0.579 |

All values are mean±SD. HF, high frequency; HRV, heart rate variability; LF, low frequency; PECO, post-exercise circulatory occlusion; pRR50, proportion of RR interval differences greater than 50 ms; RMSSD, root mean square standard deviation; SDRR, standard deviation of RR intervals. a indicates significantly different than Baseline.

**Supplemental Table 2.** The influence of OC on HRV response to arm or leg passive movement (PM).

|  | Passive Movement | | | | | | | | | | | | | | |
| --- | --- | --- | --- | --- | --- | --- | --- | --- | --- | --- | --- | --- | --- | --- | --- |
|  | Arm | | | | | | |  | Leg | | | | | | |
|  | OC | | NOC | | P-value | | |  | OC | | NOC | | P-value | | |
|  | Baseline | PM | Baseline | PM | OC | Time | OC  X Time |  | Baseline | PM | Baseline | PM | OC | Time | OC  X Time |
| SDRR (ms) | 70±34 ^a^ | 63±30 ^a,b^ | 96±38 | 87±38 ^b^ | **0.040** | **0.029** | 0.766 |  | 77±43 | 70±35 ^b^ | 92±36 | 78±28 ^b^ | 0.349 | **<0.001** | 0.219 |
| RMSSD (ms) | 84±50 | 76±52 ^b^ | 123±61 | 113±55 ^b^ | 0.052 | **0.022** | 0.719 |  | 89±64 | 80±62 ^b^ | 120±57 | 99±51 ^b^ | 0.240 | **<0.001** | 0.076 |
| pRR50 (%) | 51±24 | 45±25 ^b^ | 63±23 | 59±24 ^b^ | 0.125 | **0.029** | 0.805 |  | 51±24 | 45±26 ^b^ | 63±23 | 55±24 ^b^ | 0.183 | **0.002** | 0.684 |
| LF (nu) | 24±18 | 27±17 | 19±12 | 21±11 | 0.228 | 0.345 | 0.807 |  | 23±19 | 34±20 ^b^ | 18±12 | 23±13 ^b^ | 0.134 | **0.003** | 0.254 |
| HF (nu) | 75±17 | 70±20 | 79±12 | 76±9 | 0.249 | 0.193 | 0.642 |  | 76±19 | 65±19 ^b^ | 79±12 | 73±13 ^b^ | 0.275 | **0.004** | 0.377 |
| LF/HF ratio | 0.44±0.64 | 0.52±0.50 | 0.27±0.22 | 0.30±0.17 | 0.091 | 0.611 | 0.785 |  | 0.60±1.36 | 0.69±0.62 | 0.26±0.26 | 0.35±0.24 | 0.132 | 0.598 | 0.988 |

All values are mean±SD. HF, high frequency; HRV, heart rate variability; LF, low frequency; PM, passive movement; pRR50, proportion of RR interval differences greater than 50 ms; RMSSD, root mean square standard deviation; SDRR, standard deviation of RR intervals. a indicates significantly different than NOC. b indicates significantly different than Baseline.

**Supplemental Table 3.** Cardiorespiratory changes from baseline to arm or leg PECO and PM considering covariate analysis adjusting for the influence of muscle volume or strength.

|  |  |  | |  | ANCOVA | | | | | | | | | |
| --- | --- | --- | --- | --- | --- | --- | --- | --- | --- | --- | --- | --- | --- | --- |
|  |  |  |  |  | Analysis of Variance for Equal Slopes Model | | | | | | | | | |
|  |  | Unadjusted Means±SD | |  | Model Fit | | Adjusted Means ± Std. Err. | |  | | p-value (ηp^2^) | | | |
|  |  | OC | NOC |  | R^2^ | Adj. R^2^ | OC | NOC | |  | | OC use | Muscle Volume | Muscle  Strength |
| **Arm PECO** | ΔMAP (mmHg) | 10±8 | 8±8 |  | 0.406 | 0.334 | 12±2 ^a^ | 6±2 | |  | | **0.038** (0.16) | 0.070 (0.13) | **<0.001** (0.37) |
|  | ΔV_E_ (L/min) |  |  |  |  |  |  |  | |  | | *Assumptions for Equal Slopes Model not met* | | |
| **Leg PECO** | ΔMAP (mmHg) | 4±6 | 5±5 |  | 0.277 | 0.191 | 4±1 | 6±1 | |  | | 0.487 (0.02) | 0.409 (0.03) | **0.006** (0.26) |
|  | ΔV_E_ (L/min) | -0.009±0.7 | 0.6±1.3 |  | 0.161 | 0.0638 | 0.05±0.3 | 0.5±0.3 | |  | | 0.221 (0.06) | 0.313 (0.04) | 0.273 (0.05) |
| **Arm PM** | ΔMAP (mmHg) | 2±2 | 1±2 |  | 0.171 | 0.0711 | 2±1 | 1±1 | |  | | 0.139 (0.09) | 0.210 (0.06) | 0.768 (0.004) |
|  | ΔV_E_ (L/min) | 0.6±1.7 | 1.0±1.6 |  | 0.0348 | -0.0765 | 0.6±0.5 | 1.0±0.5 | |  | | 0.599 (0.01) | 0.563 (0.01) | 0.719 (0.005) |
| **Leg PM** | ΔMAP (mmHg) | 1±2 | 2±3 |  | 0.116 | 0.0143 | 1±1 | 2±1 | |  | | 0.276 (0.05) | 0.549 (0.01) | 0.164 (0.07) |
|  | ΔV_E_ (L/min) | 1.1±0.9 | 1.2±1.1 |  | 0.262 | 0.170 | 1.2±0.3 | 1.1±0.2 | |  | | 0.949 (0.0002) | 0.051 (0.15) | **0.045** (0.16) |

ADJ, adjusted; ANCOVA, analysis of covariance; ANOVA, analysis of variance; MAP, mean arterial pressure; NOC, non-OC users; OC, oral contraceptives; V_E_, ventilation; Raw data is presented as mean±SD and adjusted means are presented as mean±SE. Bolded text represents a significant difference (p<0.05). a indicates significantly different than NOC.
